# Hospitalizations in Australian children with neuroblastoma: A population‐based study

**DOI:** 10.1002/cam4.5806

**Published:** 2023-03-31

**Authors:** C. Signorelli, F. J. Schneuer, C. E. Wakefield, J. K. McLoone, T. Trahair, R. J. Cohn, N. Nassar

**Affiliations:** ^1^ Kids Cancer Centre, Sydney Children's Hospital New South Wales Randwick Australia; ^2^ School of Clinical Medicine, UNSW Medicine & Health, UNSW Sydney New South Wales Kensington Australia; ^3^ Faculty of Medicine and Health University of Sydney New South Wales Sydney Australia; ^4^ Children's Cancer Institute, Lowy Cancer Research Centre, UNSW Sydney New South Wales Kensington Australia; ^5^ Child Population and Translational Heath Research, The Children's Hospital at Westmead Clinical School New South Wales Westmead Australia

**Keywords:** Australia, children, cost, hospitalizations, prevalence

## Abstract

**Background:**

An increasing number of children diagnosed with both low‐ and high‐risk neuroblastoma are surviving. Yet, treatment can be intensive and often multimodal, especially for high‐risk neuroblastoma, resulting in significant long‐term health problems. We aimed to describe neuroblastoma survivors' pediatric hospitalizations, readmissions, and their associated costs.

**Method:**

We conducted a population‐based study of all children (<18 years) residing in New South Wales (NSW), Australia, and hospitalized with a recorded diagnosis of neuroblastoma during 2001–2020. We used linked NSW Admitted Patient Data Collection and death registration data to examine the frequency, length of stay, and readmissions following the first admission when neuroblastoma was diagnosed (i.e., the index admission), and the associated hospitalization costs by age and timing postindex admission discharge.

**Results:**

In total, 300 children (64% aged <3 years) were hospitalized for neuroblastoma over the study period. The median number of readmissions and length of stay within 2 years postdischarge were 17 (interquartile range IQR: 5.5–25) and 45.5 (IQR: 10–125) days, and median cost per child was AUD$124,058 (IQR $34,217–$264,627). Following discharge from the index admission, there were 7088 readmissions (median: 20 per child, IQR: 7–29). Fifty‐eight percent of readmissions occurred within 1‐year postdischarge, primarily due to fever, nausea, abdominal pain, and respiratory conditions.

**Conclusion:**

The burden of health problems requiring hospitalization among neuroblastoma survivors results in significant associated healthcare costs, warranting further efforts to optimize health care for neuroblastoma survivors that focuses on early intervention and long‐term monitoring.

## 1 INTRODUCTION

Neuroblastoma represents 6% of all childhood cancers globally and is the most common extracranial solid tumor.^1^ Overall survival for childhood neuroblastoma has incrementally improved in recent decades, but is still comparably lower than for most other childhood cancer diagnoses.^2^, ^3^ Improvements in survival rates are primarily attributable to the use of more intensive treatment in recent decades for high‐risk neuroblastoma, potentially leading to greater risk of late mortality and treatment‐related health conditions.^4^ Late effects, including (latent) second malignancies, cardiac, and pulmonary complications, contribute to a small decrease in long‐term survival rates among neuroblastoma survivors.^5^ Neuroblastoma survivors are eight times more likely than their siblings to develop health problems,^5^ most commonly affecting their neurological, sensory, endocrine, and musculoskeletal systems.^5^, ^6^ Many of these treatment‐related health conditions require hospitalization for their management. Compared with survivors of other childhood cancers, one Scandinavian study reported that neuroblastoma survivors were at the highest risk of hospitalization for developing subsequent health conditions.^7^

Cancer‐related health conditions can have adverse and wide‐reaching implications, potentially compounding the impact of the initial cancer diagnosis and treatment. These conditions can impact childhood cancer survivors' life circumstances, influencing their physical and mental health, potentially leading to lower educational attainment and decreased income and employment.^8^ In neuroblastoma survivors, chronic conditions have been shown to be associated with poorer overall health and social difficulties that can impair survivors' health‐related quality of life (HRQoL).^9^ Certain conditions such as pulmonary disease, peripheral neuropathy, and endocrine diseases in particular are associated with poor psychological health in neuroblastoma survivors.^10^, ^11^ Up to 82% of survivors also commonly report hearing loss,^6^ which has been linked to poor psychosocial and academic outcomes in school‐aged neuroblastoma survivors.^9^, ^11^

The prevalent and complex health conditions presenting in this high‐risk group are not only likely to increase healthcare expenditure, but may also lead to a greater need for support from disability programs or social welfare. Yet, the long‐term cost burden of neuroblastoma on the Australian healthcare system remains unknown, including the possible costs of unaddressed or late presenting health conditions that could be mitigated through survivorship care.

The available evidence on the health and hospitalizations of neuroblastoma survivors is also limited by reporting on cohorts in previous decades who primarily died from their primary cancer,^10^ are single‐institution studies,^12^ have modest sample sizes,^12^, ^13^ focus on specific treatments or toxicities,^12^, ^13^, ^14^, ^15^ report only on specific subgroups (e.g., survivors who were diagnosed at a certain age, or with advanced disease),^5^, ^15^, ^16^ or rely primarily on self‐reported data, increasing the potential for ascertainment and recall bias. Neuroblastoma survivors' outcomes are also often masked in larger studies reporting on the overall childhood cancer survivor cohort. Australian studies have been limited to neuroblastoma survivors treated with autologous stem cell transplantation,^15^ or focused only on second malignancies.^2^ Yet, Australian survivors experience unique challenges in survivorship, including the distance many face to travel to cancer treatment and survivorship centres,^17^ which may lead to survivors delaying care or presenting with more advanced conditions.^17^, ^18^ We therefore aimed to use population‐based data to describe pediatric hospitalizations and readmissions in children with neuroblastoma, and their associated costs. Improving our understanding of hospital service use by children diagnosed with neuroblastoma will help to inform the delivery of future models of care for this high‐risk population.

## 2 METHODS

We conducted a record‐linkage cohort study including all children (<18 years) who were diagnosed with neuroblastoma in New South Wales (NSW), Australia between January 2001 and June 2020.

### 2.1 Data sources

We obtained health information from the NSW Admitted Patient Data Collection (APDC), emergency department presentations from the NSW Emergency Department Data Collection (EDDC), and Death Registrations from the Register of Birth Deaths and Marriages (RBDM). The APDC is a census of all public and private hospital admissions that includes demographic, clinical, and health service information for each admission. A primary diagnosis and procedure and up to 50 fields of diagnoses and procedures are recorded for each hospital admission and coded according to the 10th revision of the International Classification of Diseases, Australian Modification (ICD‐10‐AM), and the Australian Classification of Health Interventions (ACHI), respectively. The EDDC is a statutory collection of emergency department presentations across all public hospitals in NSW. The NSW Centre for Health Record Linkage probabilistically linked APDC, EDDC, and Death registrations and provided de‐identified data to researchers. We obtained ethics approval for data access from the NSW Population and Health Services Research Ethics Committee (2019/ETH1153229).

Children with neuroblastoma were identified from hospital data using a recorded diagnosis of ICD10‐AM: M9500, M9522, M9490, and M9504 (codes ending in/6 indicated metastatic neuroblastoma) in any hospital admission from birth. We defined the index admission as the first in which a diagnosis of neuroblastoma was recorded. Readmissions were defined as any hospital admission occurring after discharge from the index admission. We assessed hospitalizations by evaluating the number of readmissions and cost recorded for each child up to the end of the study period, June 2020, or their death date. We categorized readmissions by timing postindex admission (within 2 years, between two and 4 years, and 5 years post). We identified reasons for readmissions using the primary diagnosis and categorized them using major ICD10‐AM diagnosis chapters (e.g., Diseases of the circulatory system: ICD10‐AM codes I00–I99; Diseases of the digestive system: K00–K99). For each diagnostic chapter, we reported the overall number, and up to five of the most common conditions identified. For readmissions with a recorded primary diagnosis in the neoplasm chapter (C00‐D49), we evaluated and reported the most common primary procedures. We also identified if the readmissions were planned or involving ED based on ED status information recorded in each admission.

We calculated costs using the Australian Refined Diagnostic Related Group (AR‐DRG) codes assigned to each admission in APDC and ED presentation in EDDC. To avoid double counting, only ED presentations not requiring admission were included in ED cost calculations. The cost of each readmission or ED presentation in $AUD was assigned using the average cost of individual AR‐DRG for the 2018–19 financial year.^19^

### 2.2 Statistical analysis

We used descriptive statistics to assess socio‐demographic, hospital, and clinical characteristics of children in the index admission. Survival rates with a 95% confidence interval were determined using Kaplan–Meir methods. We also described hospitalizations by age (<18 months, 18 months to <5 years, and ≥5 years), total readmissions by major diagnostic chapter, and timing postdischarge of index admission, following recent revisions to the age cutoff for the classification of high‐risk disease from 12 months to 18 months.^20^, ^21^, ^22^ Kruskal–Wallis test was used to evaluate differences in health utilization. We evaluated the proportion of children returning to hospital for readmissions for different primary diagnoses and their timing postdischarge, stratifying by age groups.

## 3 RESULTS

We identified 349 children aged less than 18 years who were hospitalized with neuroblastoma in NSW during 2001–2020. We excluded 49 children residing out of NSW at the time of the index admission, primarily patients living in the Australian Capital Territory and traveling to Sydney for treatment. Of the remaining 300 children, 53% (*n* = 159) were male (Table 1). Children primarily resided in major cities (*n* = 238, 79.3%). Nearly half (*n* = 129, 43%) were aged between 18 months and 5 years at the index admission. The most common site of the neuroblastoma was the adrenal gland (*n* = 123, 41%). Almost half (*n* = 138, 46%) had metastatic disease at index admission, with one‐third having bone marrow involvement. The overall one‐year survival rate was 92% (95% CI = 89%–95%), which declined to 78% (95% CI = 72%–83%) and 75% (95% CI 69%–80%) at 5 and 10 years, respectively. Survival was higher in children aged <18 months (92%; 95% CI = 87%–97%) than those aged between 18 months and less than 5 years (5‐year survival rate: 70%: 95% CI = 62%–79%) and those older or equal to 5 years (61%; 95% CI = 45%–77%).

**TABLE 1:** Demographic and clinical characteristics of children diagnosed with neuroblastoma at their index admission in New South Wales, 2001–2020.

Demographic and clinical characteristic	*N* = 300
Year of index admission^a^	*n* (%)
2001–2004	55 (18.3)
From 2005	245 (81.7)
Age at index admission	
<18 months	122 (40.7)
18 months – <5 years	129 (43.0)
≥5 years	49 (16.3)
Sex	
Male	159 (53)
Female	141 (47)
Child country of birth	
Australia	289 (96.3)
Other	11 (3.7)
Remoteness of residence	
Major cities	238 (79.3)
Inner regional	43 (14.3)
Outer regional/remote	18 (6)
Socio‐economic disadvantage quintile	
1 (Most disadvantaged)	73 (24.3)
2–4	165 (55)
5 (Least disadvantaged)	61 (20.3)
Health insurance	
Public patient	184 (61.3)
Private patient	115 (38.3)
Primary tumor site	
Adrenal gland	123 (41)
Unspecified abdomen site	33 (11)
Peritoneum	24 (8)
Peripheral nerves	21 (7)
Connective and soft tissue	15 (5)
Urinary system	14 (4.7)
Central nervous system	10 (3.3)
Other sites	42 (14)
Metastatic neuroblastoma	
No	162 (54)
Yes	138 (46)
Secondary tumor site	
Bone marrow	94 (31.3)
Lymph nodes	53 (17.7)
Other sites	51 (17.0)

*Note*: Percentages may not add to 100 due to missing values.

^a^ Year of index admission is divided into <2005 and =>2005 to align with notable changes to treatment for neuroblastoma, particularly the cessation of the use of total body irradiation.

### 3.1 Hospitalizations

The median number of readmissions and length of stay within 2 years postdischarge of the index admission was 17 (IQR: 5.5–25) and 45.5 (IQR: 10–125) days per child, respectively, and decreased to medians between 0 and 3 readmissions and/or days in the periods following (Table 2). Nearly one in five children (*n* = 41; 18%) had 30 or more readmissions in the initial follow‐up period. Children between 18 months and <5 years at their index admission had higher median number of readmissions within the next 2 years (median 20; IQR: 12–29), compared with children <18 months and ≥5 years (median 13). Median readmissions decreased to between 0 and 2 after 2 years for all age groups. Results for length of stay followed a similar pattern, although children aged ≥5 years at their index admission had higher median length of stay (63; IQR: 8–144) than children aged <18 months in the next 2 years. The median total health system cost of admissions per child in the first 2 years postindex admission was AUD$124,058 (IQR $34,217–$264,627) including ED presentations. Costs decreased after the first 2 years following the index admission. (Table 2).

**TABLE 2:** Readmissions by timing from index admission and age in children with neuroblastoma in NSW, 2001–2020.

Age group at index admission	Within 2 years, *N* = 224^a^	Between two and 4 years, *N* = 166^a^	Five or more years, *N* = 131^a^
	Median (IQR)	Median (IQR)	Median (IQR)
Number of readmissions	*p* = 0.01	*p* = 0.2	*p* = 0.2
<18 months	13 (4–23)	2 (0–5.5)	1 (0–3)
18 months – <5 years	20 (12–29)	1 (0–4)	0 (0–3)
≥5 years	13 (2–23)	1 (0–2)	1 (1–4)
Total	17 (5.5–25)	1 (0–4)	1 (0–3)
Number of ED presentations not requiring admission	*p* = 0.03	*p* = 0.5	*p* = 0.8
<18 months	2 (0–5)	1 (0–2)	1 (0–3)
18 months – <5 years	1 (0–2)	0 (0–2)	1 (0–4)
≥5 years	1 (0–2)	1 (0–1)	2 (0–3)
Total	1 (0–3)	1 (0–2)	1 (0–3)
LOS (days) of readmissions	*p* < 0.001	*p* = 0.5	*p* = 0.05
<18 months	21 (7–61)	2 (0–7)	1 (0–3)
18 months ‐ <5 years	99 (22–164)	1 (0–5)	0 (0–3)
≥5 years	63.5 (8–144)	1 (0–5.5)	3 (1–16)
Total	45.5 (10–125)	1 (0–5)	1 (0–4)
Readmission cost (AU$ ‘000)^b^	*p* < 0.001	*p* = 0.4	*p* = 0.05
<18 months	79.8 (22.5–180.8)	5.4 (0–32.2)	0 (0–8.4)
18 months – <5 years	206.7 (69.5–298.4)	5.5 (0–31.4)	0 (0–13.8)
≥5 years	125.7 (16.2–264.2)	2.6 (0–11.2)	8.6 (1.5–34.6)
Total	122.9 (33.4–263.9)	5.0 (0–28.8)	1.5 (0–13.7)
Total cost (AU$ ‘000)^b^	*p* < 0.001	*p* = 0.4	*p* = 0.08
<18 months	81.4 (26.3–183.2)	6.8 (0.8–33.5)	1.8 (0–11.4)
18 months – <5 years	207.8 (70.6–298.4)	6.6 (0–31.3)	2.2 (0–14.3)
≥5 years	126.5 (16.7–264.8)	3.2 (0.2–11.4)	8.6 (2.5–34.6)
Total	124.0 (34.2–264.6)	5.7 (0.1–30.8)	3.3 (0–14.3)

*Note*: *p*‐values for Kruskal–Wallis Test.

Abbreviations: ED, emergency department; IQR, interquartile range; LOS, length of stay.

^a^ Children with sufficient follow‐up time (children included in the 5 or more years column had at least 8 years of follow‐up time).

^b^ The exchange rate at the time of the analysis (April 2021) was $1 AUD = $0.78 USD.

In total, there were 7088 readmissions following discharge from the index admission primarily occurring within 2 years postdischarge of index admission (*n* = 5469, 77%). Ten percent of admissions (*n* = 698) occurred 5 years or more postindex admission. In the first 2 years, three quarters of readmissions were for therapy and diagnostics, while <10% were for other reasons including myelosuppression and symptoms such as fever, nausea, and abdominal pain (Table 3). The proportion of readmissions for therapy and diagnostics remained high over time (62% up to 5 years and 56% after 5 years). Most readmissions in the first 2 years were planned (*n* = 5812; 83%); however, up to 20% of readmissions involved the ED after 5 years postindex.

**TABLE 3:** Number of readmissions to hospital following the index admission in children with neuroblastoma by type (principal diagnosis or procedure) and timing in NSW, 2001–2020.

Diagnosis or procedures requiring readmission	Within 2 years, *N* = 5469		Between two and 4 years *N* = 930		Five or more years, *N* = 689		Total, *N* = 7088	
	*n* (%)^a^	%^b^	*n* (%)^a^	%^b^	*n* (%)^a^	%^b^	*n* (%)^a^	%^b^
Therapy or diagnostics	4023	73.6	578	62.2	386	56.0	4987	70.4
Pharmacotherapy and other noninvasive interventions	1331 (33.1)	24.3	142 (24.6)	15.3	63 (16.5)	9.1	1536 (31.7)	21.7
Imaging services	1150 (28.6)	21.0	267 (46.2)	28.7	15 (3.9)	2.2	1432 (29.5)	20.2
Procedures on bone marrow	423 (10.5)	7.7	43 (7.4)	4.6	19 (5)	2.8	485 (10)	6.8
Radiation oncology procedures	418 (10.4)	7.6	34 (5.9)	3.7	‐	‐	454 (9.4)	6.4
Hemodialysis	‐	‐	‐	‐	242 (63.5)	35.1	242 (5)	3.4
Other	701 (17.4)	12.8	92 (15.9)	9.9	45 (11.7)	6.5	838 (16.8)	11.8
Symptoms	346	6.3	56	6.0	36	5.2	438	6.2
Fever	246 (71.1)	4.5	25 (44.6)	2.7	7 (19.4)	1.0	278 (63.5)	3.9
Abdominal pain, nausea, or vomiting	40 (11.6)	0.7	15 (26.8)	1.6	12 (33.3)	1.7	67 (15.3)	0.9
Syncope and collapse or convulsions	13 (3.8)	0.2	5 (8.9)	0.5	‐	‐	20 (4.6)	0.3
Other	47 (13.6)	0.9	11 (19.6)	1.2	15 (41.7)	2.2	73 (16.7)	1.0
Diseases of the blood and immune system	370	6.8	34	3.7	30	4.4	434	6.1
Agranulocytosis	279 (75.4)	5.1	19 (55.9)	2.0	11 (36.7)	1.6	309 (71.2)	4.4
Thrombocytopenia	53 (14.3)	1.0	8 (23.5)	0.9	7 (23.3)	1.0	68 (15.7)	1.0
Anemia	33 (8.9)	0.6	7 (20.6)	0.8	12 (40)	1.7	52 (12)	0.7
Other	5 (1.4)	0.1	‐	‐	‐	‐	5 (1.2)	0.1
Diseases of the respiratory system	109	2.0	84	9.0	44	6.4	237	3.3
Acute upper respiratory infection	51 (46.8)	0.9	11 (13.1)	1.2	5 (11.4)	0.7	67 (28.3)	0.9
Interstitial lung disorder	‐	‐	50 (59.5)	5.4	17 (38.6)	2.5	67 (28.3)	0.9
Acute lower respiratory infection/pneumonia	31 (28.4)	0.6	12 (14.3)	1.3	8 (18.2)	1.2	51 (21.5)	0.7
Other	27 (24.8)	0.5	11 (13.1)	1.2	14 (31.8)	2.0	52 (21.9)	0.7
Diseases of the nervous system	162	3.0	42	4.5	9	1.3	213	3.0
Myoclonus	149 (92)	2.7	29 (69)	3.1	‐	‐	178 (83.6)	2.5
Obstructive sleep apnea syndrome	‐	‐	5 (11.9)	0.5	‐	‐	12 (5.6)	0.2
Cerebral palsy or paralysis	5 (3.1)	0.1	‐	‐	‐	‐	10 (4.7)	0.1
Other	‐	‐	6 (14.3)	0.6	‐	‐	13 (6.1)	0.2
Infectious and parasitic diseases	180	3.3	19	2.0	11	1.6	210	3.0
Septicemia or bacterial infection	102 (56.7)	1.9	6 (31.6)	0.6	‐	‐	108 (51.4)	1.5
Infectious gastroenteritis	39 (21.7)	0.7	8 (42.1)	0.9	6 (54.5)	0.9	53 (25.2)	0.7
Unspecified viral infection	22 (12.2)	0.4	‐	‐	‐	‐	25 (11.9)	0.4
Other	17 (9.4)	0.3	‐	‐	‐	‐	24 (11.4)	0.3
Injury, external causes, or complications from medical interventions	105	1.9	16	1.7	24	3.5	145	2.1
Complication from medical devices, implants, or grafts	78 (74.3)	1.4	‐	‐	6 (25)	0.9	88 (60.7)	1.2
Wounds or fractures	17 (16.2)	0.3	6 (37.5)	0.6	15 (62.5)	2.2	38 (26.2)	0.5
Other	10 (9.5)	0.2	6 (37.5)	0.6	‐	‐	19 (13.1)	0.3
Diseases of the digestive system	55	1.0	39	4.2	41	6.0	135	1.9
Caries or other dental conditions	14 (25.5)	0.3	18 (46.2)	1.9	16 (39)	2.3	48 (35.6)	0.7
Intussusception/intestinal obstruction/constipation	19 (34.5)	0.3	9 (23.1)	1.0	18 (43.9)	2.6	46 (34.1)	0.6
Gastrointestinal hemorrhage	10 (18.2)	0.2	11 (28.2)	1.2	6 (14.6)	0.9	27 (20)	0.4
Other	12 (21.8)	0.2	‐	‐	‐	‐	14 (10.4)	0.2
Diseases of the genitourinary system	29	0.5	19	2.0	33	4.8	81	1.1
Urinary tract infection	16 (55.2)	0.3	‐	‐	13 (39.4)	1.9	32 (39.5)	0.5
Kidney disease	10 (34.5)	0.2	11 (57.9)	1.2	10 (30.3)	1.5	31 (38.3)	0.4
Conditions of female genital organs	‐	‐	‐	‐	7 (21.2)	1.0	7 (8.6)	0.1
Other	‐	‐	5 (26.3)	0.5	‐	‐	11 (13.6)	0.2
Endocrine, nutritional, and metabolic diseases	29	0.5	‐	‐	20	2.9	53	0.8
Disorders of calcium or magnesium metabolism	10 (34.5)	0.2	‐	‐	‐	‐	11 (20.8)	0.2
Volume depletion	8 (27.6)	0.1	‐	‐	‐	‐	8 (15.1)	0.1
Other	11 (37.9)	0.2	‐	‐	19 (95)	2.8	34 (64.2)	0.5
Diseases of the skin and subcutaneous tissue	17	0.3	11	1.2	‐	‐	32	0.4
Diseases of the eye and adnexa	9	0.2	‐	‐	13	1.9	25	0.4
Diseases of the ear and mastoid process	20	0.1	13	1.4	6	0.9	39	0.4
Diseases of the musculoskeletal system and connective tissue	9	0.1	‐	‐	12	1.7	24	0.3
Congenital anomalies or perinatal	8	0.2	‐	‐	7	1.0	19	0.3
Diseases of the circulatory system	7	0.1	5	0.5	5	0.7	17	0.2
Mental and behavioral disorders	‐	‐	‐	‐	8	1.2	10	0.1

*Note*: ^a^Percentage within the group; ^b^Overall percentage; − denotes <5. NB: The numbers in rows may not add up to totals as we have not presented results where there were <5 cases to preserve confidentiality.

**FIGURE 1:**
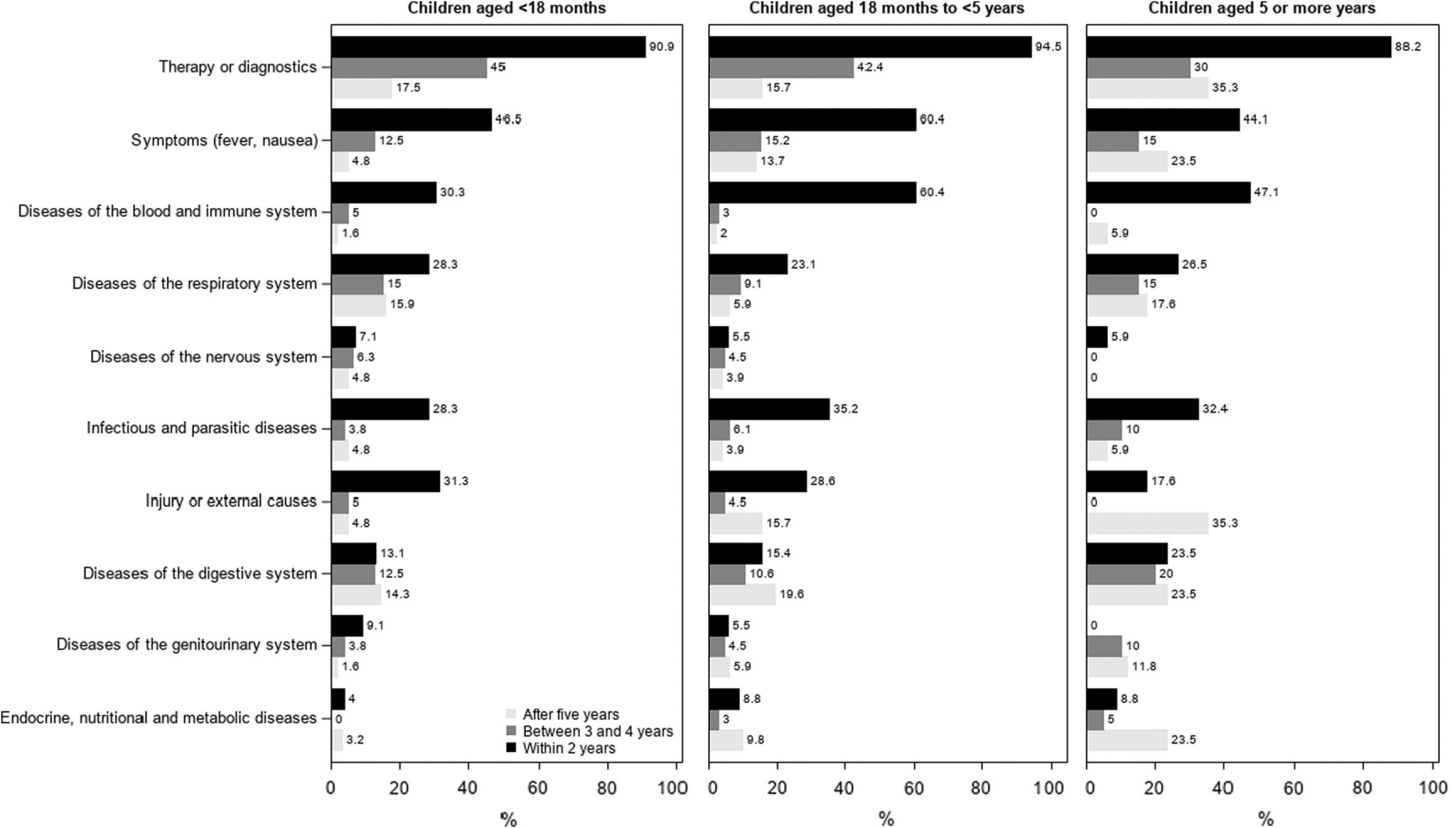
Proportion of children admitted to hospital by major ICD10‐AM diagnosis chapters (principal diagnosis or procedure) and timing postindex admission for children with neuroblastoma in NSW, 2001–2020. Results represent percentages of survivors with at least one readmission and with sufficient follow‐up time (children included in the 5 or more years bar had at least 8 years of follow up time).

The proportion of children readmitted to hospital by diagnostic category is presented in Figure 1. Most children of all age groups (between 88% and 95%) were readmitted for therapy and diagnostics within 2 years postdischarge of the index admission. The proportion of children admitted for therapy or diagnostics decreased over time following the index admission. In the first 2 years following the index admission, children aged between 18 months and 5 years were more likely to be admitted for symptoms (60%) and diseases of the blood and immune system (60%), compared with children aged <18 months (47% and 30%) and ≥5 years (44% and 47%). Other common reasons for readmissions within 2 years following the index admissions were infectious or parasitic diseases (between 28% and 35% of children), diseases of the respiratory system (between 23% and 28%), and injury or external causes (between 18% and 31%) (Figure 1).

## 4 DISCUSSION

This population‐based study investigated pediatric hospitalizations among children with neuroblastoma both during and postcancer treatment. We identified 300 children who were hospitalized for treatment of neuroblastoma, equating to approximately 15 children per year. These children had a median of 20 admissions to hospital, most of which were planned. Over half occurred within 1 year of the index admission. The median total health system costs of these admissions were AUD$124,058 per child in the first 2 years postindex admission and significantly decreased thereafter. The most common reasons for readmission to hospital in the first 5 years postindex admission were related to therapy and diagnostics followed by symptoms such as fever, nausea, and abdominal pain followed by diseases of the blood and immune system. One in 10 readmissions occurred after 5 years postindex admission.

The median number of hospital admissions in children with neuroblastoma appears to be higher compared with another population‐based analysis of children diagnosed with any cancer (3.9 average admissions during a 10‐year period), or in children with other chronic conditions (1.6 admissions).^23^ Around three quarters of readmissions to hospital in our cohort were recorded in the first 2 years postindex, which may reflect commonly experienced side effects of cancer treatment and related acute symptoms (e.g., nausea related to chemotherapy and abdominal pain related to radiotherapy). It seems that our data more likely reflect the experiences of high‐risk rather than nonhigh‐risk neuroblastoma patients, with admissions aligning with the expected rate reflected by therapy (including chemotherapy, surgical resection, high dose consolidation therapy with autologous stem cell rescue, and potentially radiation therapy) delivered as inpatient care and within the first 12–15 months postdiagnosis. Respiratory conditions were among the highest recorded within 5 years postindex admission, which another study showed to be the most costly, alongside hospitalization for neurological conditions (e.g., ear conditions and epilepsy).^23^ Where possible, it is vital to minimize these hospitalizations given their impact not only on the healthcare system, but to the child and the whole family unit. For example, young children hospitalized with a chronic condition are vulnerable to poorer developmental outcomes (e.g., socially, physically, and emotionally), and school‐aged children are more likely to underperform academically in both literacy and numeracy domains if they experience frequent hospitalizations.^24^, ^25^

Hospitalizations for some conditions were generally low in our cohort even for commonly reported conditions in neuroblastoma survivors (e.g., cardiovascular conditions), possibly not capturing all of the care provided for nonhigh‐risk neuroblastoma patients or primarily lower grade toxicities which are managed in the outpatient setting. Over the studied period, treatment for nonhigh‐risk neuroblastoma has steadily de‐intensified in this cohort without compromising survival,^26^, ^27^, ^28^ and the standard of care for high‐risk neuroblastoma has increased with the introduction of anti‐GD2 therapy (requiring five inpatient admissions)^29^, ^30^ and the inclusion of tandem transplantation.^31^ The survival rates reported in our cohort align with international evidence,^21^ although are higher compared with other Australian data,^2^ likely reflecting the older period of that study.

The nature and diversity of conditions in survivors in our study, coupled with up to 20% of readmissions requiring emergency department visits, reinforce the need for ongoing surveillance and comprehensive survivorship care to monitor for treatment‐related conditions. Treatment for neuroblastoma substantially impacts survivors' long‐term health, with evidence suggesting poorer outcomes both physically and psychosocially.^5^ In a US study, survivors with at least one severe or life‐threatening condition were more likely to report poorer overall health, greater bodily pain, and were more likely to be unemployed.^9^ A UK study demonstrated that neuroblastoma survivors have among the highest excess risks for day patient hospitalizations, compared with survivors of other childhood cancers.^32^ Yet, increased engagement in specialized survivorship care has been shown to be associated with a decrease in emergency department visits and hospitalizations.^33^ Alongside increased primary care involvement to manage the growing population of survivors, early screening and intervention may help to mitigate emergency department visits, length of hospital stays, and thereby the associated costs.

Some reasons for admissions in our sample indicate survivors' risk of other health problems; for example, metabolic symptoms may reveal diabetes, or digestive symptoms may reflect malabsorption. Some persistent conditions (e.g., pain and fatigue) in isolation might not appear clinically significant, but may pose a substantial burden to survivors and cause long‐term physical and psychological suffering.^34^ There are ongoing international trials that have prioritized improving our knowledge of less common—yet still personally and clinically significant—health problems in neuroblastoma survivors.^35^ Studies have also shown that neuroblastoma survivors have a higher prevalence of chronic conditions compared with siblings and controls,^5^, ^9^ particularly for pulmonary, auditory, gastrointestinal, neurological, and renal conditions.^9^ The ongoing development, and increasing implementation of patient‐reported outcome measures in research and clinical practice, may help patients to voice some of the symptoms relating to these conditions with their cancer care team or family physician. These measures may facilitate early intervention, thereby preventing hospitalizations, including emergency department visits, and reducing associated costs to the patient and healthcare system. This is particularly important given that admissions for chronic conditions in children account for almost half of total hospital costs.^23^

### 4.1 Study limitations

Data linkage is the most feasible way to access a longitudinal population‐based perspective of health outcomes and healthcare use that is not subject to recruitment or recall bias.^2^, ^36^ However, our focus on hospitalization data may reflect the more severe spectrum of health conditions that children with neuroblastoma face in survivorship and did not capture conditions which do not require hospitalization, although they may still be significant or life altering. Another limitation is that we did not have information on patients that moved out of state and were lost to follow‐up or those who received treatments in hospitals in other states. In our study, we could not elicit specific treatment data or information about the stage of each survivors' cancer, although we can infer that patients with metastases (46% of our sample) were likely to be stage 4. Without available treatment data, we relied on survivors' age at their index admission (<18 months vs. >18 months) as a proxy for level of risk, which is important to consider given the distinct biology of low‐risk, compared with high‐risk neuroblastoma.^2^ Future studies should systematically evaluate the incidence and nature of late effects in neuroblastoma survivors, particularly considering the evolving treatments for neuroblastoma in recent decades. These limitations warrant further research investigating the needs of specific subgroups of neuroblastoma patients (e.g., stratified by stage of cancer, risk level, specific treatment(s) received, and their doses) and compared with children without a history of cancer.

## 5 CONCLUSIONS

Treatment for neuroblastoma is often intensive and multimodal, particularly for high‐risk patients, resulting in significant long‐term health conditions. A considerable number of hospitalizations were recorded in our cohort, reflecting conditions impacting multiple systems that can severely impact survivors' quality of life. Long‐term monitoring and intervention are essential to reduce the prevalence of these conditions and their substantial cost to the patient and healthcare system. Given the potential burden of health problems requiring hospitalization on survivors' long‐term physical and psychosocial health, further effort is warranted in optimizing healthcare for neuroblastoma survivors that focuses on patient education and early intervention.

## AUTHOR CONTRIBUTIONS

**Christina Signorelli:** Conceptualization (equal); funding acquisition (lead); investigation (equal); methodology (equal); project administration (lead); resources (equal); writing – original draft (lead); writing – review and editing (lead). **Francisco J Schneuer:** Data curation (lead); formal analysis (lead); investigation (equal); methodology (lead); software (lead); visualization (lead); writing – original draft (supporting); writing – review and editing (equal). **Claire Wakefield:** Conceptualization (lead); investigation (supporting); project administration (supporting); supervision (equal); writing – review and editing (equal). **Jordana McLoone:** Conceptualization (supporting); investigation (supporting); methodology (supporting); writing – review and editing (supporting). **Toby Trahair:** Conceptualization (supporting); investigation (supporting); writing – review and editing (supporting). **Richard Cohn:** Conceptualization (supporting); investigation (lead); supervision (equal); writing – review and editing (lead). **Natasha Nassar:** Conceptualization (lead); data curation (lead); formal analysis (supporting); investigation (supporting); methodology (equal); project administration (supporting); supervision (equal); writing – review and editing (lead).

## FUNDING INFORMATION

This study was supported by Neuroblastoma Australia. Christina Signorelli is supported by Neuroblastoma Australia and a Cancer Institute NSW Early Career Fellowship (2020/ECF1144). Claire E. Wakefield is supported by the National Health and Medical Research Council of Australia (APP2008300). Natasha Nassar is funded by the Financial Markets Foundation for Children and the National Health and Medical Research Council of Australia (APP1197940).

## CONFLICT OF INTEREST STATEMENT

No author has any conflicts of interest to disclose in relation to this manuscript.

## Data Availability

The data that support the findings of this study are not publicly available due to privacy or ethical restrictions, and the full dataset is not able to be released due to ethical restrictions. Requests may be made to the authors.
